# Efficient and Parallel Implementation of Real and Complex Response Functions Employing the Second-Order Algebraic-Diagrammatic Construction Scheme for the Polarization Propagator

**DOI:** 10.1021/acs.jctc.3c01065

**Published:** 2023-12-20

**Authors:** Manuel Brand, Andreas Dreuw, Patrick Norman, Xin Li

**Affiliations:** † Division of Theoretical Chemistry and Biology, School of Engineering Sciences in Chemistry, Biotechnology and Health, KTH Royal Institute of Technology, Stockholm SE-100 44, Sweden; ‡ Interdisciplinary Center for Scientific Computing, Ruprecht-Karls University, Im Neuenheimer Feld 205, Heidelberg 69120, Germany; § PDC Center for High Performance Computing, KTH Royal Institute of Technology, Stockholm SE-100 44, Sweden

## Abstract

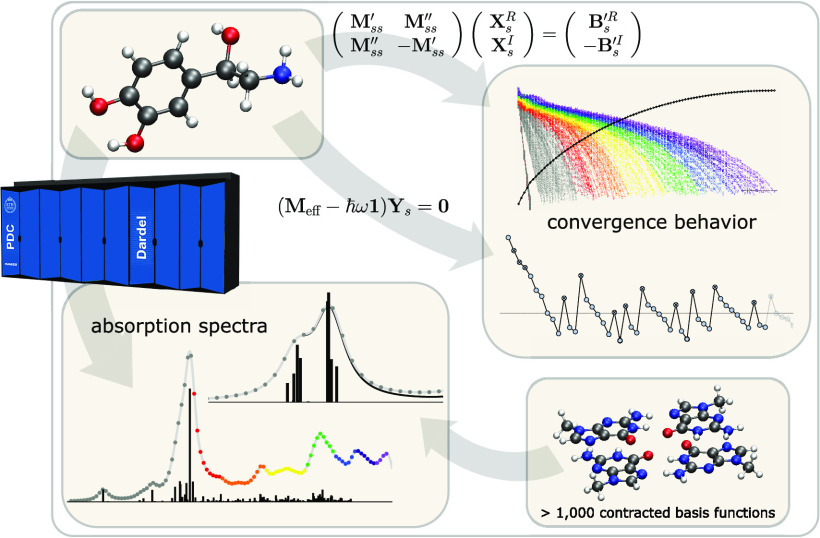

We present the implementation of an efficient matrix-folded formalism for the evaluation of complex response functions and the calculation of transition properties at the level of the second-order algebraic-diagrammatic construction (ADC(2)) scheme. The underlying algorithms, in combination with the adopted hybrid MPI/OpenMP parallelization strategy, enabled calculations of the UV/vis spectra of a guanine oligomer series ranging up to 1032 contracted basis functions, thereby utilizing vast computational resources from up to 32,768 CPU cores. Further analysis of the convergence behavior of the involved iterative subspace algorithms revealed the superiority of a frequency-separated treatment of response equations even for a large spectral window, including 101 frequencies. We demonstrate the applicability to general quantum mechanical operators by the first reported electronic circular dichroism spectrum calculated with a complex polarization propagator approach at the ADC(2) level of theory.

## 1 Introduction

The calculation of molecular properties and spectra by means of quantum chemical methods has become an integral part of all molecular sciences. Ranging from the elucidation of reaction mechanisms to trend predictions for properties of nanomaterials, theoretical chemistry offers a pathway to reach a fundamental understanding of the problem at hand based on first-principles, revealing insights that are often not accessible through experimental setups.^1,2^ Arguably, even more than established experimental techniques, the adopted theoretical methods must be scrutinized with respect to accuracy and reliability for any given application. While density-functional theory (DFT) and time-dependent DFT in many cases yield accurate results at relatively low computational costs, the fact that these methods cannot be systematically improved leads to problems in cases when they fail to adequately describe the electronic structure.^3,4^ Methods that do provide a way toward systematic improvements of results are therefore of heightened importance, and besides coupled cluster (CC) approaches, the algebraic-diagrammatic construction (ADC) scheme for the polarization propagator has shown strong merits with a good balance between accuracy and computational cost.^5,6^

The ADC formalism was first introduced up to a second-order perturbative treatment (ADC(2)) in 1982^7^ and was later extended to third-order (ADC(3))^8−10^ and recently to fourth-order (ADC(4)).^11^ An alternative derivation known as the intermediate state representation (ISR)^12,13^ enabled the treatment of open-shell systems,^14^ the calculation of excited state and transition properties,^15^ one-electron and detachment/attachment densities,^16−18^ as well as linear and higher-order response properties,^19−23^ making ADC a versatile method. For instance, the combination of ADC with response theory has led to calculations of *C*_6_ dispersion coefficients of the electronic ground and excited states^24,25^ and resonant inelastic X-ray cross sections,^26^ showing excellent agreement with theoretical benchmark values and experimentally observed quantities, respectively.

In the past, second- and third-order ADC and CC methods have typically served as benchmarks for cheaper alternatives. However, the recent advances made in the area of high-performance computing (HPC) have put us at the doorstep of exascale computing, and from a pure floating-point operation point of view, it ought to be possible to employ such methods more routinely for systems of real technical and/or biochemical interest. Contemporary supercomputers can provide access to thousands of compute nodes, each hosting beyond a hundred cores, as exemplified by the LUMI system within the EuroHPC Joint Undertaking project.^27^ To efficiently harness such massively parallel supercomputers for response property, or spectroscopy, calculations employing electron-correlated wave function methods is notoriously difficult and often introduces memory and/or communication bottlenecks. In the case of ADC, the implementation in the Q-Chem program^28^ is parallelized with the hybrid open multiprocessing (OpenMP) scheme for single-node execution. The Turbomole program^29,30^ implements a parallelization based on a hybrid OpenMP/message-passing interface (MPI) scheme in conjunction with the resolution-of-identity (RI) approximation for the evaluation of electron repulsion integrals.^31^ The Psi4 program^32^ implements an interface to the OpenMP-parallel ADC-connect (adcc) module for single-node execution and thereby provides a variety of ADC excited-state calculations.^33^

More recently, the Gator program^34^ was released. Alongside the aforementioned adcc module, it contains the Respondo module for the evaluation of real and complex linear response functions and the HPC-QC module implementing ADC(2) with a hybrid MPI/OpenMP parallelization scheme that features an integral transformation that is driven by an efficient and massively parallel construction of auxiliary Fock matrices using the VeloxChem program.^35^ It was demonstrated that excitation energies could be determined for systems involving some 800 contracted basis functions without introducing any integral approximations.

In the present work, the functionality of this module is extended to encompass the calculation of transition moments as well as real and complex linear response functions as defined in the complex polarization propagator (CPP) framework^36,37^ and with an adopted matrix-folding technique to avoid explicit reference to the two-electron transfer amplitudes of response vectors.^38^ Some practical implications of the underlying formalism—derived for ADC(2) in ref (39)—are discussed, and the capabilities of the implementation are demonstrated by calculations of the ultraviolet/visible absorption (UV/vis) and circular dichroism (CD) spectra for noradrenaline and the UV/vis spectra for a series of guanine oligomers.

## 2 Method and Implementation

The original derivation of ADC via many-body Green’s function theory and its ISR reformulation have been summarized elsewhere at different levels of detail.^7,8,12,40,41^ The key equations for the latter are briefly introduced in the following for self-containment.

Central to the ADC formalism stands the Hermitian eigenvalue equation
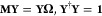
1with the ADC matrix **M**, the matrix of eigenvectors **Y**, and the diagonal eigenvalue matrix **Ω** containing the excitation energies *ℏ*ω_0*n*_. Hereby, the ADC matrix corresponds to the representation of the Hamiltonian shifted by the ground-state energy  in the basis of the intermediate states 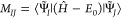
2In general, these states are explicitly constructed from a correlated ground-state |Ψ̃_0_⟩ by acting on it with a set of electron excitation operators
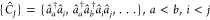
3where, respectively,  and  are the canonical creation and annihilation operators for a general orbital p and indices *i*, *j*, ... and *a*, *b*, ... refer to occupied and unoccupied orbitals in the associated Hartree–Fock state. By means of these particle–hole (*p*–*h*), two-particle–two-hole (2*p*–2*h*), *etc.* excitations, a correlated excited-state basis is formed. The resulting states are subsequently orthogonalized by employing a Gram–Schmidt procedure to yield the intermediate state basis. In case of ADC, the *n*th-order Møller–Plesset (MP) ground-state wave function and energy^42^ in the perturbative expansions
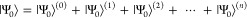
4
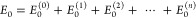
5serve as the starting point to arrive at the *n*th-order scheme ADC(*n*).

The ISR derivation of ADC allows for a straightforward transformation of time-dependent response functions into closed-form matrix expressions.^19,20^ A general frequency-dependent linear response function is given as
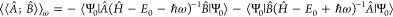
6Inserting the resolution of identity of the intermediate states  yields the ADC formulation of the linear response function
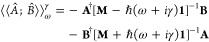
7where the modified transition moments  as well the imaginary damping terms *i*γ are introduced. The latter ensure resonance-convergence of the response functions and account for the finite lifetime of excited states. In practice, a common γ is typically adopted for all excited states.^43,44^

Each of the terms in eq 7 can be evaluated by means of the CPP approach. Taking the first term as an example, a complex response vector **X** is to be determined by solving the complex linear equation
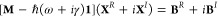
8To avoid complex algebra, eq 8 may be expressed as a coupled set of equations involving a real symmetric matrix
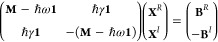
9In the case of ADC(2), the 2*p*–2*h*/2*p*–2*h* block of the matrix **M** is only expanded up to the zeroth order of perturbation theory^40^ and hence exhibits diagonality. It is therefore advantageous to exploit the underlying block matrix structure to express the doubles excitation parts of the response vector **X**_*d*_^*R*^ and **X**_*d*_^*I*^ by means of their singles excitation counterparts **X**_*s*_^*R*^ and **X**_*s*_^*I*^. This *matrix folding* procedure, outlined in ref (39), yields a matrix equation that is solely expressed in blocks of the size of the *p*–*h* manifold
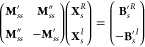
10where the indices *ss* and *s* refer to the singles excitation manifold-size of matrix and vector blocks, respectively. Equation 10 can subsequently be solved using an iterative subspace algorithm in order to avoid the computational effort caused by inversion of the folded ADC matrix. The subspace is spanned by a set of real so-called trial vectors
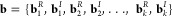
11that are used in the linear transformations σ^′^ = **M**_*ss*_^′^**b** and σ^″^ = **M**_*ss*_^″^**b**. As a consequence of the folding, the blocks **M**_*ss*_^′/″^(ω) are frequency-dependent, resulting in the same property for the sets of **σ**′^/^″ vectors
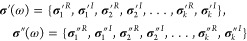
12With the diagonal 2*p*–2*h*/2*p*–2*h* block considered implicitly, the implementation of the algebraic expressions for the computationally demanding construction of the vectors σ_*i*_^′^, σ_*i*_^″^, and **B**_*s*_^′^ employs an hybrid MPI/OpenMP integral transformation.^34^ Thereby, auxiliary Fock matrices
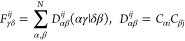
13are used to express the electron-repulsion integrals in the molecular orbital (MO) basis
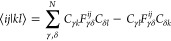
14where *i*, *j*, *k*, *l* and α, β, γ, δ denote the MO and atomic orbital (AO) indices, respectively, *N* is the number of basis functions, and density matrices *D* are contractions of MO coefficient matrices *C*. In this way, the integrals may be constructed incrementally, and a highly parallelized routine provided by the VeloxChem program allows for the construction of whole batches of auxiliary Fock matrices with a single evaluation of the necessary integrals in the AO basis. Furthermore, usage of a split MPI communicator not only enables the matrix construction to be carried out across cluster nodes but also results in the MO integrals being stored on the node of their evaluation, optimizing the usage of aggregated memory of the available nodes, significantly reducing internodal communication, and making the integrals readily available for the distributed calculation of the aforementioned components. Additionally, the computed vectors are stored and the operations
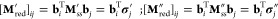
15
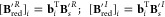
16are carried out across all available cluster nodes. Previously, the latter has been successfully applied for the calculation of static polarizabilities and *C*_6_ coefficients at the DFT level of theory.^45^ With the elements from eqs 15 and 16, the reduced space response equation
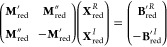
17can be constructed and solved with negligible computational effort. Accordingly, the optimal solution vectors in the *n*th iteration are obtained by
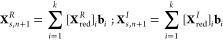
18Upon convergence of the subspace algorithm, the desired response property requires multiplication of the left-hand side transition moments **A**^†^ with the response vector **X** in the full *p*–*h* and 2*p*–2*h* manifold
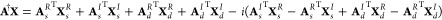
19While the terms of the form **A**_*s*_^T^**X**_*s*_ can be calculated directly, the doubles excitation vector products are accessible through the expressions for **X**_*d*_^*R*/*I*^ used for the folding of the matrix equation. The respective combinations of real and imaginary components lead to explicit expressions that are calculated at a low cost from the node-distributed MO integrals.

The second term in eq 7 can be written as the complex conjugate of the first term with the negative frequency
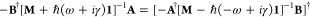
20and can hence be computed in the same way by replacing ω with −ω and taking the complex conjugate of the result for the calculation of the molecular response property of interest. As a consequence, the evaluation of each response function involves solving two reduced space matrix equations.

The implementation includes simultaneous handling of a large set of optical frequencies as well as multiple right-hand sides **B** for calculations of discretized spectra. For that, a user-defined option allows to choose between the usage of either a common set of trial vectors for all frequency-dependent subspaces or a separate set for every individual frequency. A detailed discussion about the effect on the performance and convergence behavior of this choice is presented in Section 3.

Ultimately, the evaluation of complex response functions gives rise to the quantities of the linear absorption cross section σ(ω) and anisotropy of the decadic molar extinction coefficient Δ*ϵ*(ω), defining UV/vis and CD spectra, respectively, via
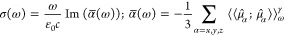
21where  is the electric-dipole operator along the Cartesian coordinate α in the molecular frame, and
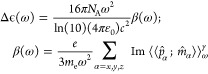
22where *p̂* and *m̂* are the linear momentum and magnetic dipole operators, respectively.^46^

Analogously to the CPP equation, the ADC(2) equation in eq 1 for a specific eigenvalue may be folded into an expression only including matrices and vectors with the size of the singles excitation manifold
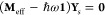
23where the effective ADC(2) matrix **M**_eff_ exhibits a dependence on ω, making the eigenvalue problem nonlinear. Similarly to the procedure outlined above, a reduced space block Davidson algorithm^47^ may be applied to obtain a solution for the eigenvalues and -vectors. Unlike in the evaluation of the CPP equation, however, the frequency ω is not given, and a nonlinear solver needs to be employed. Thereby, updated eigenvalues are determined with the Newton–Raphson method according to
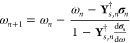
24where the contraction
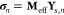
25and its derivative with respect to the eigenvalue
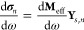
26are the central computational steps in every iteration *n*.

An augmentation of the folded ADC(2) solver implemented in the HPC-QC module of the Gator program^34^ capable of calculating a manifold of transition properties via contraction of the converged eigenvectors with the respective modified transition moments
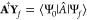
27is included in this work. The latter directly give rise to, e.g., oscillator and rotatory strengths of the corresponding transition 0 → *f*.

The workflow of the latest implementation of the eigenvalue solver is briefly outlined in Section 3, together with a presentation of its convergence characteristics.

## 3 Convergence Behavior

**Figure 1:**
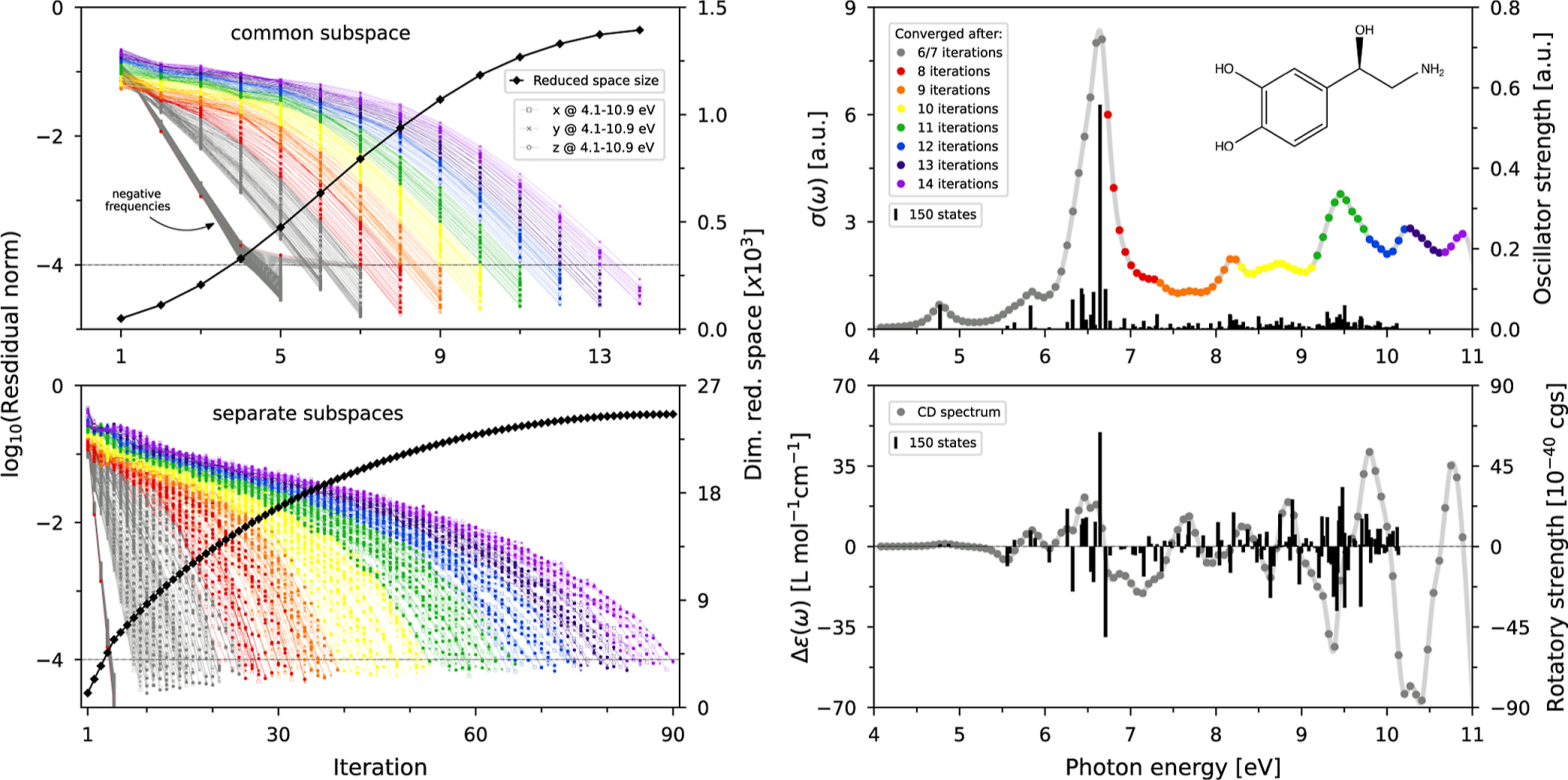
Convergence behavior of the CPP implementation quantified by the norm of the residual vectors and the amount of spawned trial vectors for a common subspace construction (top left) and separate subspace constructions (bottom left). The resulting UV/vis spectrum of (*R*)-noradrenaline (top right) is color-coded by the number of iterations needed for convergence using a common subspace construction. The UV/vis as well as the CD spectrum (bottom right) are complemented by the oscillator/rotatory strengths of the lowest 150 excited states. The geometry used for the calculations was optimized at the B3LYP/cc-pVTZ level of theory and the damping parameter γ in the CPP procedure was set to a value of 1000 cm^–1^.

In order to investigate the convergence behavior of the folded CPP solver implementation, the UV/vis spectrum of (*R*)-noradrenaline was calculated with two alternative procedures for subspace construction. The choice of the system in combination with the aug-cc-pVDZ basis set designed for post-Hartree–Fock methods^48,49^ is motivated by its reasonable size of 375 contracted basis functions, the presence of a stereogenic center that qualifies for a CD signal, and the successful use for testing the convergence behavior of a similar implementation at the level of single determinant approximation methods.^35^ In the spectral region between 4 and 11 eV, a total of 606 complex response equations were solved for 101 equidistant frequencies. The left panels in Figure 1 depict the norm of the residual vector of every response function until convergence at a relative threshold value of 10^–4^, together with the corresponding accumulated number of spawned trial vectors in every iteration.

As mentioned in Section 2, two different strategies for subspace construction for each frequency have been implemented. In the first, the subspace for all response equations is generated from a common set of trial vectors, all of which have been orthonormalized with respect to each other. This strategy has previously led to a major improvement of the convergence behavior in the case of single determinant approximation methods.^35^ However, as can be readily seen from eq 12, the folding of the ADC matrix introduces a dependency of the **σ**-vectors on the frequency, making it necessary to construct one pair of **σ**′/**σ**″ per frequency. Therefore, a second option enables the construction of separate subspaces, where the evaluation at every frequency is carried out with an individual set of trial vectors used to spawn the respective **σ**-vectors. From the comparison between the two alternatives, the expected benefit in terms of required iterations and spawned vectors from pooling the trial vectors in a common subspace is evident: a mere number of 1394 trial vectors generated in 14 iterations is to be compared to a total of 24,597 trial vectors in 90 iterations. Furthermore, the color-coded values for the number of iterations needed for the last converged component necessary for the determination of the linear absorption cross section in the UV/vis spectrum (top right panel) reveal the strictly progressive convergence of the spectral window included in the calculation. Hereby, a similar convergence pattern is obtained with the separate subspace procedure.

However, the frequency dependency of **σ**′^/^″(ω) is not taken into account by the presented metrics. While the separate subspace procedure is characterized by the equivalence of the number of trial vectors and constructions of **σ**-vector pairs, the common subspace procedure exhibits a more complicated correlation. The number of **σ**′/**σ**″-constructions involved in the latter is given by the number of trial vectors *times* the number of unconverged response functions in every iteration. As a result, a total of 153,380 vector pairs were constructed for the chosen frequency window—6.2 times more compared to the separate subspace procedure, associated with a 1.8-fold higher computational effort. The discrepancy between those scaling factors arises from the efficiency gain of computing larger batches of **σ**-vectors per frequency.

It should be mentioned that the implementation strategy using separate subspaces for every individual frequency still includes the handling of multiple right-hand sides per subspace, and the identical loop-structure to the common subspace strategy implies that all response equations are solved simultaneously. The computational superiority even for the relatively large frequency window chosen in this example suggests that the majority of cases will favor separate subspace handling. However, the calculation of the oscillator strengths of the lowest 150 excited states revealed an apparent increase in the number of iterations and hence trial vectors needed for convergence as the density of states (DOS) increased in the spectral region. As an example, the response equations assigned to the frequencies marked in red and green converged within 28 and 63 iterations for corresponding DOS values of 16.5 and 55.1 states/eV, respectively. The same trend is observed for the common subspace strategy but to a much smaller extent, potentially leading to an advantage over the use of separate subspaces for spectral regions with a high DOS, as encountered in, e.g., X-ray absorption spectra.^50^

**Figure 2:**
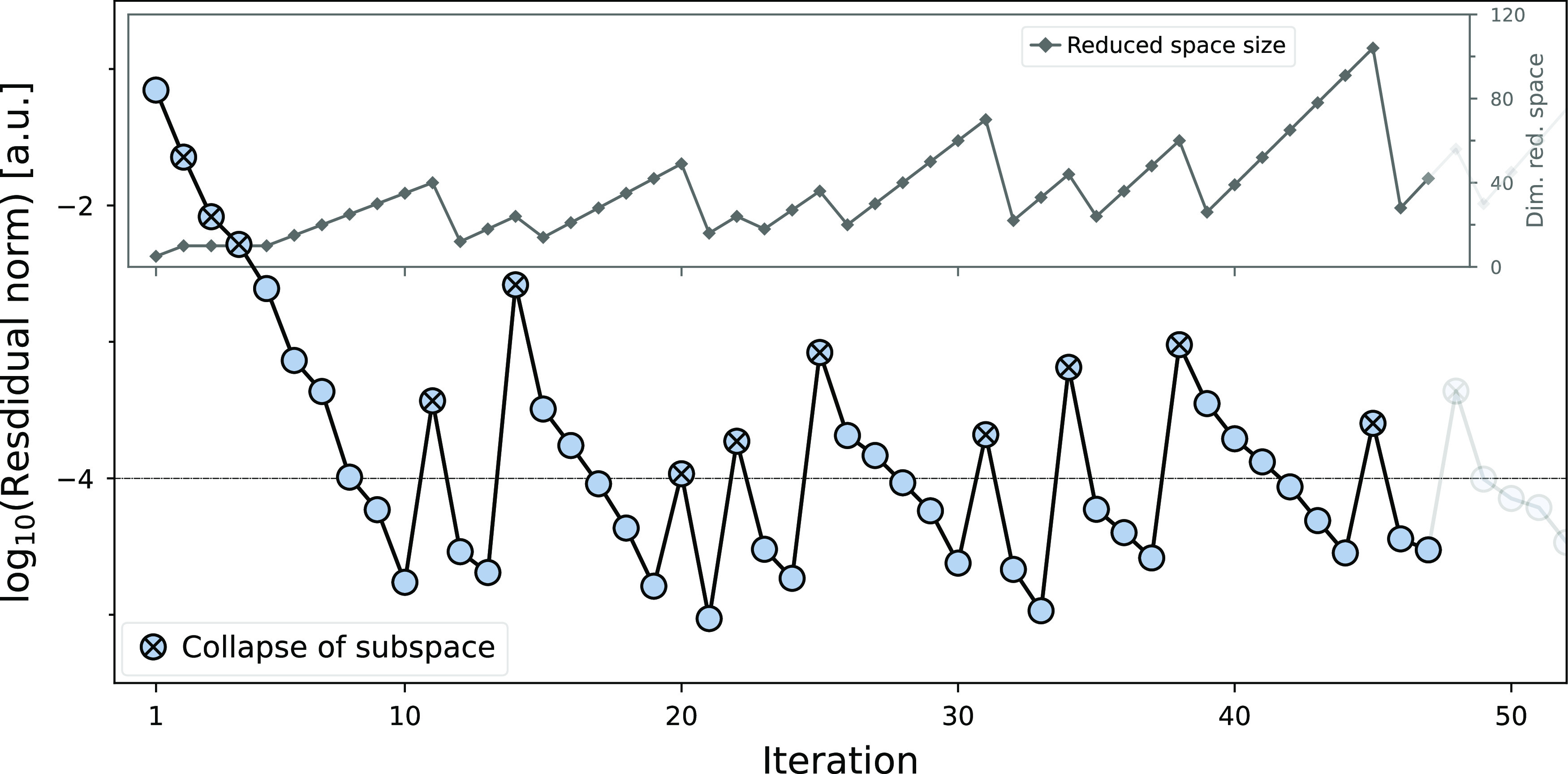
Convergence behavior of the eigenvalue solver implementation quantified by the norm of the residual vectors and the amount of spawned trial vectors for the subspace construction for the calculation of the first 10 excited states of (*R*)-noradrenaline.

The convergence characteristics of the folded nonlinear ADC(2) eigenvalue solver for the same system (*R*)-noradrenaline are shown in Figure 2. To provide a clearer picture, the norm of the residual vector and the dimension of the subspace space are indicated only for the number of iterations that led to convergence of the first 10 excited states.

**Figure 3:**
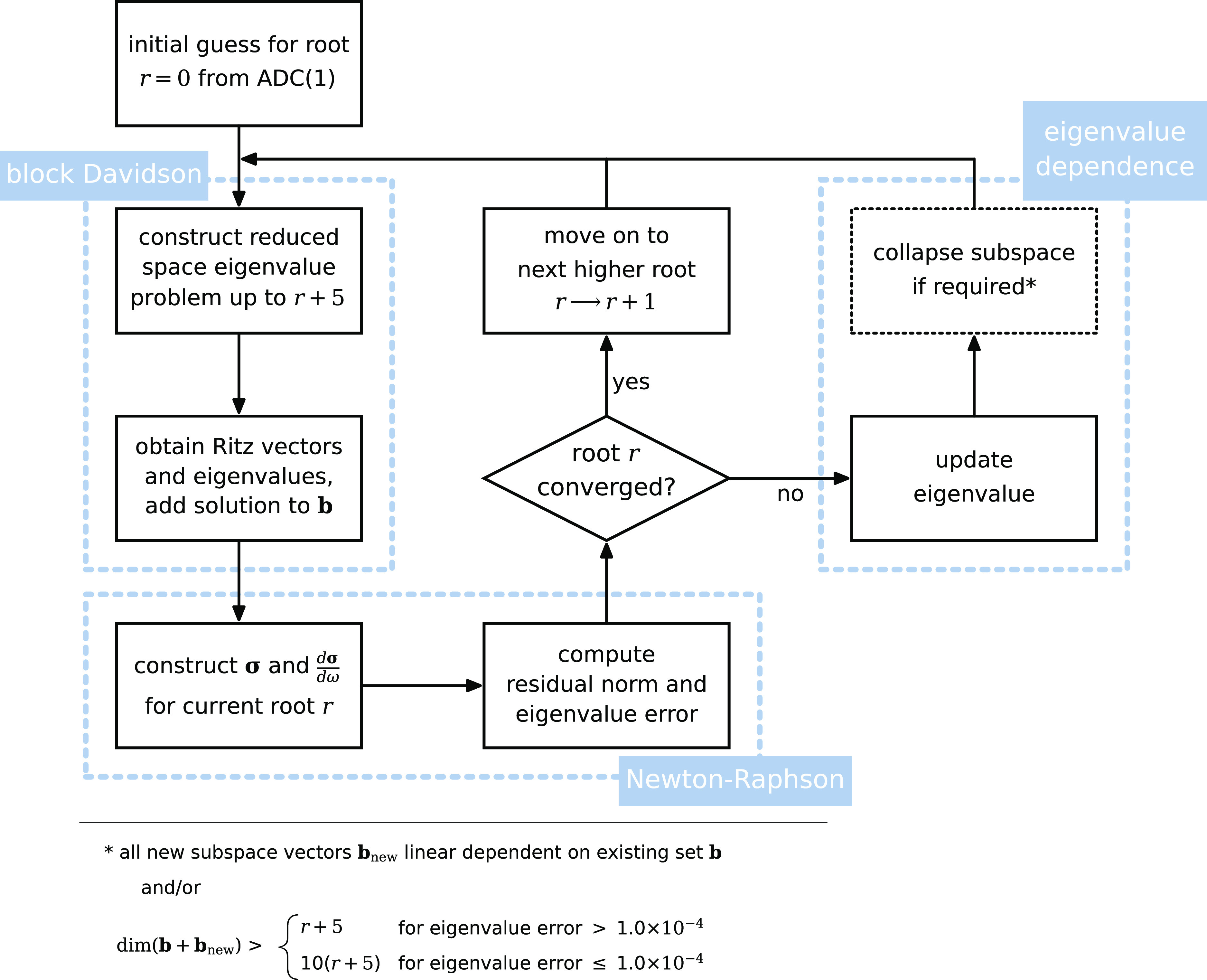
Schematic overview of the workflow of the ADC(2) eigensolver implementation.

Generally, every iteration comprises two main steps: first, the reduced space eigenvalue problem is constructed from the set of subspace vectors and solved via a block Davidson algorithm to yield Ritz vectors and values that serve as the updated solution. Second, the **σ** vector and its derivative are computed from the latter for the current root according to eqs 25 and 26 and subsequently used to produce a better approximation of the eigenvalue in a Newton–Raphson step (eq 24). While the initial set of subspace vectors and the guess for the first eigenvalue are taken from a preceding ADC(1) calculation, the updated solution from the Davidson procedure serves as the source for new subspace vectors in every iteration. To ensure that the subspace vectors provide a good representation of the eigenvalue problem of the current root and to keep the computational cost manageable, the subspace is *collapsed* under certain conditions by disregarding all previously obtained vectors and generating a new set of vectors based on the latest eigenvalue. This is done automatically either when a predefined maximum number of subspace vectors is reached (Figure 3) or no new vectors can be added due to linear dependencies on the existing set. Furthermore, subspace vectors are only generated from nonconverged eigenvectors corresponding to the next five higher eigenvalues by default. The effect of the subspace collapse and progressive incorporation of roots on the overall number of vectors used to span the subspace can be seen in the inset of Figure 2.

Along with the norm of the residual vectors, the eigenvalue error determined by the Newton–Raphson method is used as a criterion for convergence. The resulting convergence pattern for the lowest 150 excited states for (*R*)-noradrenaline is exemplified by the first 10 states, which converge within 47 iterations, while never more than 104 subspace vectors are handled at once. The consistently smooth convergence up to a high number of excited states is attested by the successful calculation of all 150 states within a total of 485 iterations, averaging to a manageable number of 335 trial vectors.

Both the implementation of the CPP solver and eigenvalue solver in the HPC-QC module are not limited to the calculation of polarizabilities and oscillator strengths, respectively, but are designed to handle any quantum mechanical operator. Currently, the functionality comprises the electric dipole, magnetic dipole, linear momentum, and angular momentum operators and arbitrary combinations thereof in the case of the CPP solver. As a demonstration, the CD spectrum of (*R*)-noradrenaline was calculated by means of the CPP approach via eq 22 along with the rotatory strengths of the lowest 150 excited states (Figure 1, bottom right panel). With the CPP solver implemented in the Respondo module being limited to the treatment of electric dipole operators, our example constitutes the first-ever CPP-CD spectrum calculated at the ADC level of theory.

## 4 Size-Scaling

A limit to the range of applications of all methods is ultimately determined by size-scaling. As hardware technologies advance and more computational resources become available, these boundaries are continuously pushed forward, given that efficient utilization is made of the resources. In the context of contemporary quantum chemical software codes, one trademark of an efficient implementation can be seen as the preservation of the size-scaling beyond the aforementioned boundaries when no further approximations are introduced. This requires the avoidance of bottlenecks caused by, e.g., memory limitations or internodal communication in the case of parallel implementations.

**Figure 4:**
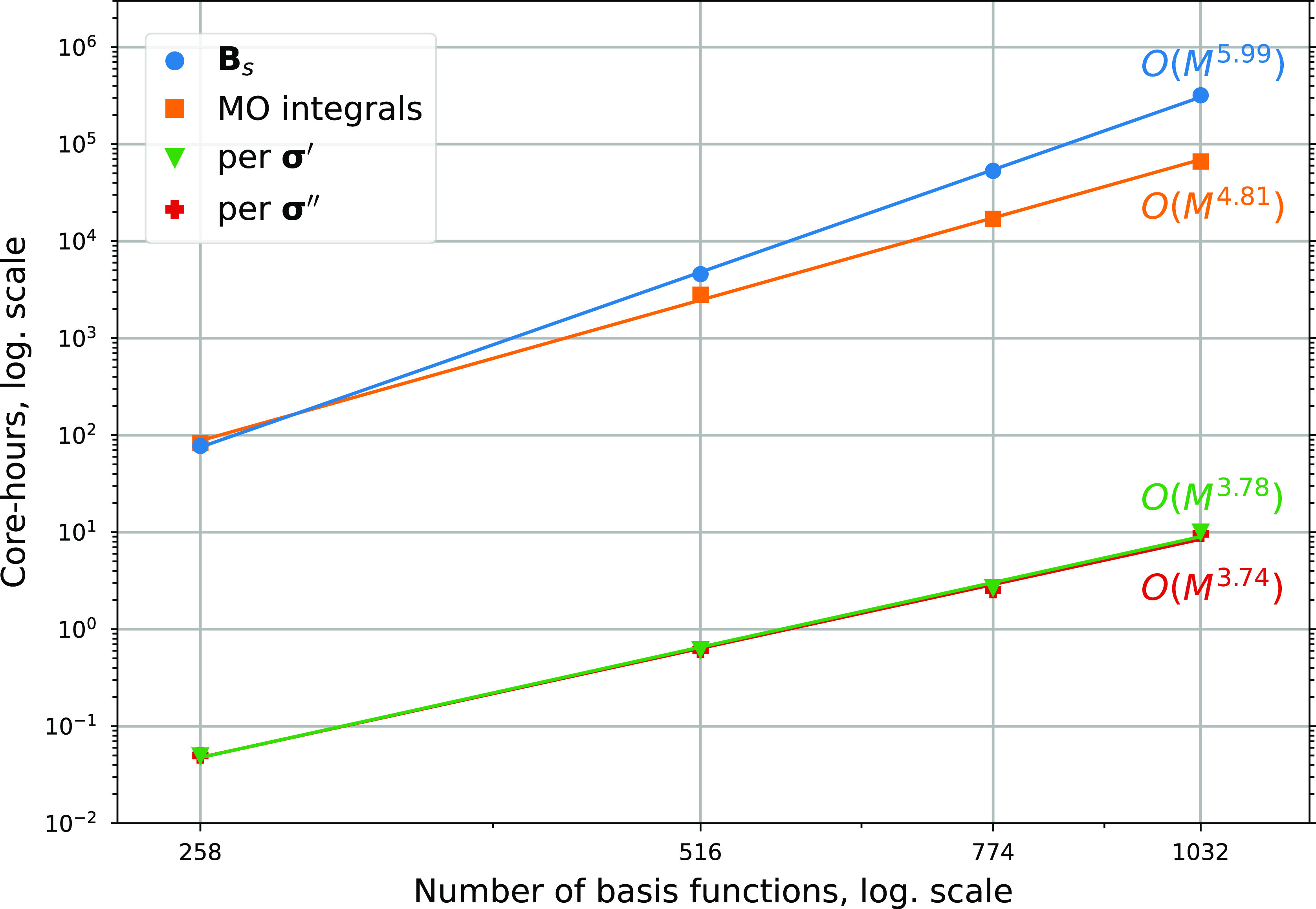
Scaling of the construction time of modified transition moments (**A**_*s*_/)**B**_*s*_, MO integrals, and the vectors **σ**′ and **σ**″ with respect to the number of contracted basis functions. The timings are obtained from calculations of a series of guanine oligomers applying the 6-311G** basis set with 258 contracted basis functions per monomeric unit. The calculations were performed on cluster nodes with a dual AMD EPYC 7742 2.25 GHz 64 core processor (256 GB) in the case of the modified transition moments and a dual AMD EPYC 7763 2.5 GHz 64 core processor (256 GB) for the MO integrals and vector contractions.

The timings and scaling with respect to the system size of the central steps in an ADC(2) excited-state calculation performed by the HPC-QC module in Gator were reported in ref (34). With the modified transition moments in the singles excitation manifold **B**_*s*_ and the matrix–vector contractions leading to the vectors **σ**′ and **σ**″, the size-scaling of three components associated with considerable computational effort has to be taken into account for the implementation work reported herein. Furthermore, the extension of the MO integrals calculation by additional integral blocks necessary for the construction of the modified transition moments requires the corresponding value obtained previously to be updated. Using the same series of guanine oligomers, but the larger 6-311G** basis set^51^ the calculations of UV/vis absorption spectra were carried out with both the CPP approach and Lorentzian broadening of oscillator strengths.^43^ The latter were made available from the extension of the ADC(2) implementation outlined in Section 2. The scalings with respect to the number of basis functions *N* shown in Figure 4 are obtained from calculations performed on the HPC-systems LUMI^27^ and Dardel.^52^

The calculation of the modified transition moments is comparable in computational cost to the construction of the MO integrals for the single guanine molecule. However, the scaling of *N*^6.0^ causes it to become more expensive than the latter for the dimer already and eventually leads to a relative computation time that is almost 5 times higher for the largest system consisting of 1032 contracted basis functions. The reported timings comprise the computation of the modified transition moments for the dipole operator in all three spatial components needed for the determination of the linear absorption cross section (eq 21). It should be mentioned that in cases for which the modified transition moments of different operators are needed (**A** ≠ **B**) or the simultaneous calculation of oscillator strengths and rotatory strengths^43^ is carried out, twice or three times as many components, respectively, are required. However, the implementation handles whole batches of operator matrices in each matrix multiplication, and hence their number does not affect the absolute computation time significantly and has no impact on the reported scaling.

The relatively high computational effort is attributed to the terms included in the perturbative second-order correction to the singles excitation manifold of the modified transition moments.^7^ By far, the largest contribution can be assigned to the term denoted *A*_*s*_^(2,9)^
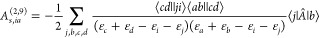
28The included two-electron integrals of the form ⟨vv||vv⟩ together with the combination of indices for the orbital energies in the denominator lead to a loop-structure with two nested loops over o,o and v,v pairs of indices, respectively (o = occupied, v = virtual). The scaling of *N*^6.0^ itself is caused by the combination of indices not only for this term but also found in 6 additional out of 13 total terms.

The constructions of the vectors **σ**′ and **σ**″ exhibit great similarity to each other and the **σ**-vectors used for solving the ADC(2) eigenvalue equation. Consequently, an equivalent scaling of *N*^3.8^ and *N*^3.7^ was found in the performed CPP calculations. Despite the much lower scaling and practically negligible absolute timing in comparison to the modified transition moments and MO integrals, a consideration of the computational effort is justified by the number of constructions executed for the calculation of the spectra evaluated at 25 different frequencies. Determined by the choice of the advantageous procedure using separate subspaces for every frequency, a total of 2374, 3043, 3457, and 3465 pairs of **σ**′- and **σ**″-vectors were constructed for the guanine oligomer series. The corresponding combined computation times amount to 316, 82, 34, and 21% of the construction of the modified transition moments, making it the overall most expensive part of the calculation for the monomer. These numbers are, however, highly individual, not only for the number of frequencies chosen for a certain calculation but also for the frequencies themselves. Generally, a response function requires a larger subspace to reach convergence in a spectral region with a high DOS. Nonetheless, the size-scaling obtained for the construction per vector is generally applicable and shows that the associated relative computational effort recedes toward system sizes of 1000 basis functions and beyond.

The scaling of *N*^4.8^ for the construction of MO integrals is in agreement with the value reported previously.

A factor that has not yet been taken into account is the number of cluster nodes that was used for the individual calculations. It is obvious from the fact that the time needed for the construction of the modified transition moments for the guanine tetramer alone would exceed 100 days if performed on a single cluster node on the Dardel system but amounts to less than 1 h for the monomer, that a different number of nodes had to be used in the presented calculations for practical reasons. With all calculations performed on nodes carrying dual AMD EPYC processors each with 64 central processing unit (CPU) cores, the ones of the modified transition moments were carried out on 4, 32, 128, and 256 nodes, amounting to 512, 4096, 16,384, and 32,768 cores, with increasing size of the oligomer series, respectively. The MO integrals and **σ** vectors were calculated on 10, 10, 80, and 160 nodes or 1280, 1280, 10,240, and 20,480 cores. Thereby, owing to the internal architecture of the CPU partition of the employed supercomputers, a division of every physical computing node into 8 MPI ranks—corresponding to the respective domains of nonuniform memory access (NUMA) —with 16 OpenMP threads each (1 per CPU core) was used, as this allocation exhibits the most efficient behavior for large-scale calculations. Although the timings presented herein stem from two different series of calculations carried out on slightly different hardware systems (Figure 4), it should be emphasized that the presented algorithms do not exhibit any multistage character per se that would render interim storage of single components in between separate calculations necessary. However, the *option* to do so enabled the reuse of the modified transition moments obtained from the series of calculations of the oscillator strengths on the Dardel system across platforms for the calculation of the CPP spectra on LUMI, leading to a lack of timings for the latter. While the chosen numbers of compute nodes for both series of calculations might seem rather odd, they were mostly governed by practical factors such as availability, expected queuing times, or total wall time limits arising from shared-user system policies.

The reported scalings with system size in combination with the absolute computing times for the various components of the implemented algorithms establish a certain range of applicability given the availability of computational resources. They do, however, not allow for a reliable assessment of the performance scaling of the parallelization of the implementation. For that, a systematic investigation toward the fulfillment of the laws of Amdahl^53^ and Gustafson^54^ would be required to determine the strong and weak scaling of the implementation, respectively. Although such an investigation lies beyond the scope of this work, the fact that a consistent size-scaling could be obtained for all central steps, despite the varying number of employed CPU cores from 512 up to 32,768 for the calculation of the modified transition moments, testifies to a degree of parallelization that is capable of utilizing a vast amount of computing power.

**Figure 5:**
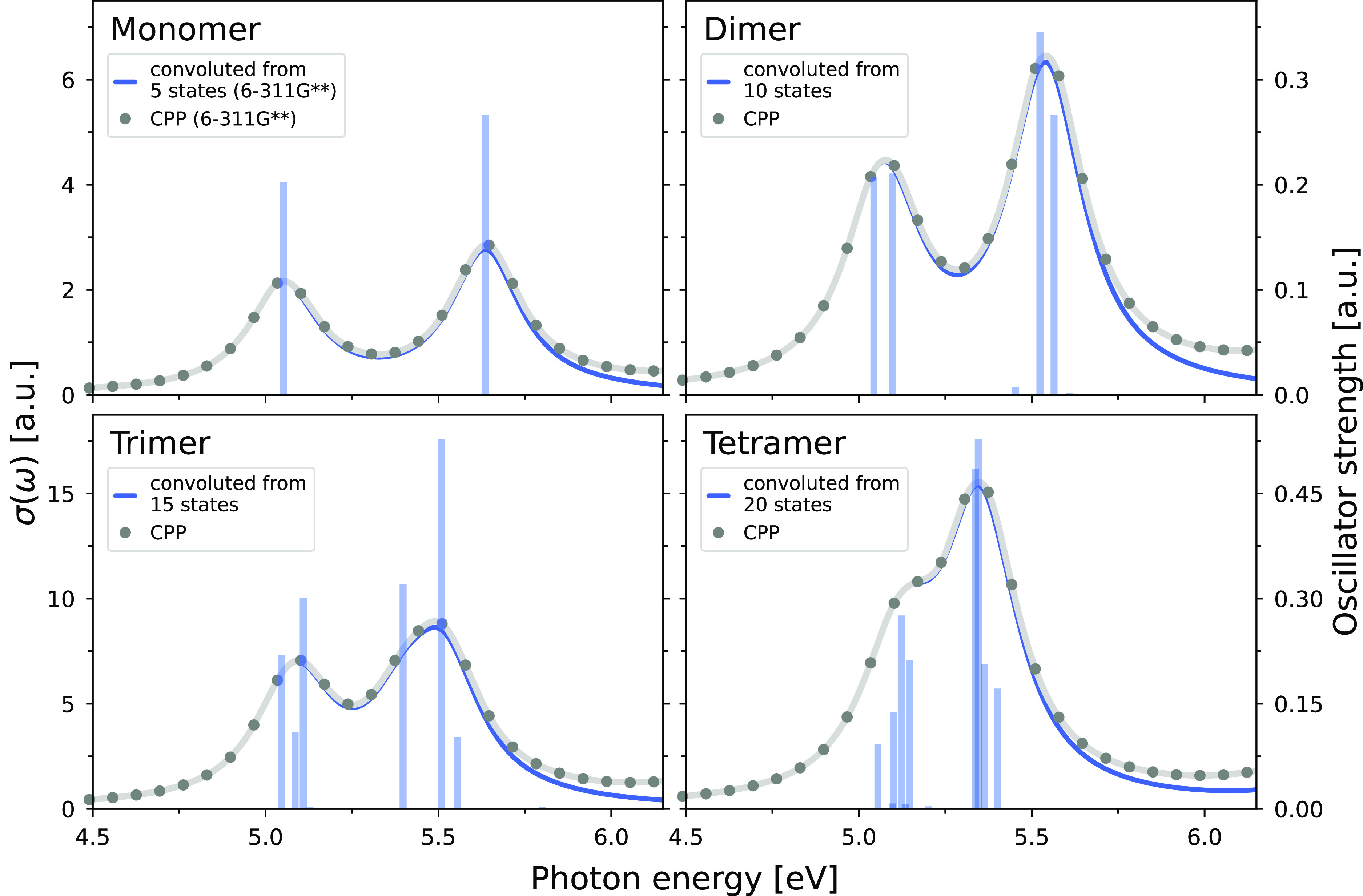
UV/vis spectra of the guanine oligomer series. A frequency window consisting of 25 frequencies (from 0.1650 to 0.2250 au in steps of 0.0025 au) was chosen for the evaluation of complex response functions using the CPP approach. The blue curves correspond to a Lorentzian line broadening of the oscillator strengths from 5, 10, 15, and 20 states, respectively. A broadening term of γ = 1000 cm^–1^ has been used for both the CPP approach and the Lorentzian line broadening.

The resulting UV/vis spectra of the calculations performed to obtain size-scalings are shown in Figure 5. In contrast to the determination of an absorption spectrum by applying a broadening function to oscillator strengths, the evaluation of a response function with the CPP approach does not suffer from the finite number of excited states considered in the calculation. The striking agreement between the spectra obtained for both techniques with a damping parameter γ of 1000 cm^–1^ indicates that the first two absorption bands are well separated in energy from all higher-lying bands, and the number of excited states calculated describe the spectral region of interest sufficiently well. It can be clearly seen that every monomeric unit contributes two bright excited states, one per absorption band. Consequently, the number of excited states contributing to each band increases from one for the monomer to four for the tetramer, although the intensity is not distributed evenly among them. Furthermore, the first absorption band remains at a constant excitation energy, while the higher-lying band experiences a red-shift with an increase in system size, causing the two bands to merge in the spectrum of the tetramer.

## 5 Summary

An implementation for the evaluation of real and complex linear response functions at the level of ADC(2) based on a combination of the CPP approach with a matrix-folding technique to avoid direct handling of matrices and vectors expressed in the doubles excitation manifold was integrated into the HPC-QC module of the Gator^34^ program package. Further implementation work was concerned with the extension of the ADC(2) eigenvalue solver to the calculation of transition properties, such as oscillator and rotatory strengths. Thereby, both implementations exploit highly efficient MPI/OpenMP-parallelized routines for a cluster node-distributed calculation and storage of two-electron MO integrals from the VeloxChem program package.^35^ The matrix equations central to the respective formalisms are solved via iterative subspace algorithms. The construction of modified transition moments and contraction of the folded ADC matrices with the trial vectors spanning the subspace is carried out across all available cluster nodes thereby utilizing the distributed MO integrals enabled by an MPI-parallelization scheme.

An investigation of the CPP solver convergence behavior of two strategies for subspace construction by means of the calculation of the UV/vis spectrum of (*R*)-noradrenaline revealed the advantage of handling the response functions for each frequency in a separate subspace over a common pooling of trial vectors. The reduced computational cost despite a larger number of iterations and trial vectors needed for convergence is substantiated by the frequency dependence of the **σ**-vectors, leading to a large number of their constructions for a common subspace. The presented example suggests that the latter might offer a suitable option for spectral regions with a high DOS, which remains to be investigated.

In the case of the eigenvalue solver, a strategy for controlled collapses of the subspace ensures a low computational cost while maintaining a smooth convergence up to a high number of excited states calculated.

The general applicability of the implementations for any quantum mechanical operator was demonstrated with the calculation of the CD spectrum of (*R*)-noradrenaline, the first ever CD spectrum calculated with the CPP approach at the ADC level of theory.

The scaling with the system size of the computationally most demanding tasks was determined by the calculation of the UV/vis spectra for a series of guanine oligomers ranging between 258 and 1032 contracted basis functions *N* for the employed 6-311G** basis set. The scalings of *N*^6.0^, *N*^4.8^, and *N*^3.8/3.7^ obtained for the computation of the modified transition moments, MO integrals, and vector pairs **σ**′/**σ**″, respectively, are in line with expectations and substantiate the capability of the implementation to handle molecular systems of relevant sizes.

The implementation work presented herein constitutes an important contribution toward the utilization of the vast computational resources that are becoming increasingly accessible, enabling a more routine use of ADC(2) as an accurate wave function-based method for applications involving up to and beyond 1000 basis functions without introducing any integral approximations.

## Supporting Information Available

Input geometries of the presented molecular property calculations (PDF)
