# Supplementary material for: Efficient and Parallel Implementation of Real and Complex Response Functions Employing the Second-Order Algebraic-Diagrammatic Construction Scheme for the Polarization Propagator
Source: J Chem Theory Comput. 2023 Dec 20;20(1):103–13. doi: 10.1021/acs.jctc.3c01065 (PMC10782457; doi:10.1021/acs.jctc.3c01065)
Supplement: Supplementary file 1 — ct3c01065_si_001.pdf [file ct3c01065_si_001.pdf]

*Supplementary information for:*

# Efficient and parallel implementation of real and complex response functions employing the second-order algebraic-diagrammatic construction scheme for the polarization propagator

Manuel Brand,[1,a] Andreas Dreuw,[2] Patrick Norman,[1] and Xin Li[3]

[1] *Department of Theoretical Chemistry and Biology, School of Engineering Sciences in Chemistry, Biotechnology and Health, KTH Royal Institute of Technology, SE-100 44 Stockholm, Sweden*

[2] *Interdisciplinary Center for Scientific Computing, Ruprecht-Karls University, Im Neuenheimer Feld 205, 69120 Heidelberg, Germany*

[3] *PDC Center for High Performance Computing, KTH Royal Institute of Technology, SE-100 44 Stockholm, Sweden*

[a] Electronic mail: manuelbr@kth.se

## INPUT GEOMETRIES FOR THE MOLECULAR PROPERTY CALCULATIONS

Geometries are given as atom type and the corresponding x, y, z components in angstrom

### (*R*)-noradrenaline

| | | | |
|---|---|---|---|
| H | -1.8193999674 | -2.5097458050 | -0.5507950455 |
| C | -1.4355452194 | -1.5284769930 | -0.2951571832 |
| C | -2.3089191215 | -0.4593458985 | -0.1886635299 |
| O | -3.6690464443 | -0.5422210733 | -0.3841138405 |
| C | -1.8255516449 | 0.8097372093 | 0.1469046451 |
| C | -0.4687604354 | 0.9845502431 | 0.3694347370 |
| H | -0.1169650009 | 1.9731850398 | 0.6359161766 |
| C | 0.4203521635 | -0.0850019011 | 0.2563051158 |
| C | 1.9048341672 | 0.1432961237 | 0.4528861030 |
| C | -0.0738036281 | -1.3439593880 | -0.0746469636 |
| H | 0.6049466138 | -2.1813113202 | -0.1482676236 |
| O | -2.6753261092 | 1.8678164911 | 0.2657837532 |
| H | -3.9133744651 | -1.4429007059 | -0.6142762974 |
| H | -3.5694739457 | 1.5581900821 | 0.0765607930 |
| O | 2.5513450005 | -0.9759063254 | 1.0721777983 |
| H | 2.0561900870 | 1.0385736809 | 1.0650086348 |
| C | 2.6349951121 | 0.3620412906 | -0.8693646417 |
| H | 2.1445626765 | 1.1787081547 | -1.4017612725 |
| N | 4.0273077512 | 0.7299013711 | -0.6192540306 |
| H | 2.5065073570 | -0.5476233229 | -1.4727537911 |
| H | 4.4869804996 | -0.0238192548 | -0.1223947057 |
| H | 4.5220904955 | 0.8575433726 | -1.4930596178 |
| H | 2.1653934540 | -1.1046663754 | 1.9436295799 |

**Guanine monomer**

| | | | |
|---|---|---|---|
| C | 23.42900 | 2.94400 | -2.72900 |
| N | 24.31900 | 2.01900 | -3.47100 |
| C | 25.18500 | 1.07300 | -2.97600 |
| N | 25.81400 | 0.43500 | -3.92300 |
| C | 25.33400 | 0.99400 | -5.09800 |
| C | 25.65000 | 0.70700 | -6.45200 |
| O | 26.45200 | -0.13600 | -6.86900 |
| N | 24.93400 | 1.50600 | -7.33500 |
| C | 24.02300 | 2.46800 | -6.97000 |
| N | 23.43100 | 3.14100 | -7.97300 |
| N | 23.72200 | 2.74300 | -5.71000 |
| C | 24.40400 | 1.98000 | -4.83400 |
| H | 22.70600 | 3.35800 | -3.40000 |
| H | 25.32200 | 0.88900 | -1.97000 |
| H | 23.65900 | 2.93200 | -8.90800 |
| H | 22.77000 | 3.84200 | -7.77200 |
| H | 25.09400 | 1.37000 | -8.31300 |
| H | 22.92700 | 2.40800 | -1.95000 |
| H | 24.01200 | 3.73300 | -2.30100 |

**Guanine dimer**

| | | | |
|---|---|---|---|
| C | 30.94600 | -5.66700 | -3.50800 |
| N | 30.49200 | -5.19300 | -4.82800 |
| C | 30.80600 | -5.61800 | -6.09300 |
| N | 30.18600 | -4.93300 | -7.02400 |
| C | 29.41400 | -4.00000 | -6.34400 |
| C | 28.52900 | -2.98300 | -6.77800 |
| O | 28.18400 | -2.63000 | -7.92100 |
| N | 27.97300 | -2.28500 | -5.70100 |

| | | | |
|---|---|---|---|
| C | 28.24100 | -2.54000 | -4.37700 |
| N | 27.62600 | -1.77900 | -3.46000 |
| N | 29.06300 | -3.48300 | -3.95400 |
| C | 29.60100 | -4.15800 | -4.98200 |
| C | 23.42900 | 2.94400 | -2.72900 |
| N | 24.31900 | 2.01900 | -3.47100 |
| C | 25.18500 | 1.07300 | -2.97600 |
| N | 25.81400 | 0.43500 | -3.92300 |
| C | 25.33400 | 0.99400 | -5.09800 |
| C | 25.65000 | 0.70700 | -6.45200 |
| O | 26.45200 | -0.13600 | -6.86900 |
| N | 24.93400 | 1.50600 | -7.33500 |
| C | 24.02300 | 2.46800 | -6.97000 |
| N | 23.43100 | 3.14100 | -7.97300 |
| N | 23.72200 | 2.74300 | -5.71000 |
| C | 24.40400 | 1.98000 | -4.83400 |
| H | 30.65900 | -4.96100 | -2.75700 |
| H | 31.45900 | -6.39200 | -6.29100 |
| H | 27.00800 | -1.06800 | -3.74500 |
| H | 27.79300 | -1.93100 | -2.50200 |
| H | 22.70600 | 3.35800 | -3.40000 |
| H | 25.32200 | 0.88900 | -1.97000 |
| H | 23.65900 | 2.93200 | -8.90800 |
| H | 22.77000 | 3.84200 | -7.77200 |
| H | 25.09400 | 1.37000 | -8.31300 |
| H | 27.33300 | -1.54600 | -5.91000 |
| H | 30.49800 | -6.61500 | -3.29600 |
| H | 32.01100 | -5.76900 | -3.51300 |
| H | 22.92700 | 2.40800 | -1.95000 |
| H | 24.01200 | 3.73300 | -2.30100 |

**Guanine trimer**

| | | | |
|---|---|---|---|
| C | 30.94600 | -5.66700 | -3.50800 |
| N | 30.49200 | -5.19300 | -4.82800 |
| C | 30.80600 | -5.61800 | -6.09300 |
| N | 30.18600 | -4.93300 | -7.02400 |
| C | 29.41400 | -4.00000 | -6.34400 |
| C | 28.52900 | -2.98300 | -6.77800 |
| O | 28.18400 | -2.63000 | -7.92100 |
| N | 27.97300 | -2.28500 | -5.70100 |
| C | 28.24100 | -2.54000 | -4.37700 |
| N | 27.62600 | -1.77900 | -3.46000 |
| N | 29.06300 | -3.48300 | -3.95400 |
| C | 29.60100 | -4.15800 | -4.98200 |
| C | 23.42900 | 2.94400 | -2.72900 |
| N | 24.31900 | 2.01900 | -3.47100 |
| C | 25.18500 | 1.07300 | -2.97600 |
| N | 25.81400 | 0.43500 | -3.92300 |
| C | 25.33400 | 0.99400 | -5.09800 |
| C | 25.65000 | 0.70700 | -6.45200 |
| O | 26.45200 | -0.13600 | -6.86900 |
| N | 24.93400 | 1.50600 | -7.33500 |
| C | 24.02300 | 2.46800 | -6.97000 |
| N | 23.43100 | 3.14100 | -7.97300 |
| N | 23.72200 | 2.74300 | -5.71000 |
| C | 24.40400 | 1.98000 | -4.83400 |
| C | 30.21300 | -5.13000 | -15.07300 |
| N | 29.46200 | -4.11800 | -14.29400 |
| C | 28.67600 | -3.08500 | -14.73800 |
| N | 28.16400 | -2.38400 | -13.76200 |
| C | 28.64600 | -2.99800 | -12.61000 |
| C | 28.42100 | -2.67100 | -11.24600 |

| | | | |
|---|---|---|---|
| O | 27.72800 | -1.74100 | -10.80900 |
| N | 29.08300 | -3.52900 | -10.38100 |
| C | 29.86600 | -4.57800 | -10.80600 |
| N | 30.42500 | -5.29800 | -9.82700 |
| N | 30.08700 | -4.89900 | -12.07600 |
| C | 29.44800 | -4.07000 | -12.92500 |
| H | 30.65900 | -4.96100 | -2.75700 |
| H | 31.45900 | -6.39200 | -6.29100 |
| H | 27.00800 | -1.06800 | -3.74500 |
| H | 27.79300 | -1.93100 | -2.50200 |
| H | 22.70600 | 3.35800 | -3.40000 |
| H | 25.32200 | 0.88900 | -1.97000 |
| H | 23.65900 | 2.93200 | -8.90800 |
| H | 22.77000 | 3.84200 | -7.77200 |
| H | 30.40500 | -5.98400 | -14.45700 |
| H | 28.50700 | -2.88300 | -15.73600 |
| H | 31.00000 | -6.06600 | -10.04800 |
| H | 30.26200 | -5.06000 | -8.88600 |
| H | 25.09400 | 1.37000 | -8.31300 |
| H | 27.33300 | -1.54600 | -5.91000 |
| H | 28.98600 | -3.37800 | -9.39700 |
| H | 30.49800 | -6.61500 | -3.29600 |
| H | 32.01100 | -5.76900 | -3.51300 |
| H | 29.63500 | -5.42600 | -15.92300 |
| H | 31.14100 | -4.71100 | -15.40100 |
| H | 22.92700 | 2.40800 | -1.95000 |
| H | 24.01200 | 3.73300 | -2.30100 |

**Guanine tetramer**

| | | | |
|---|---|---|---|
| C | 30.94600 | -5.66700 | -3.50800 |
| N | 30.49200 | -5.19300 | -4.82800 |
| C | 30.80600 | -5.61800 | -6.09300 |
| N | 30.18600 | -4.93300 | -7.02400 |
| C | 29.41400 | -4.00000 | -6.34400 |
| C | 28.52900 | -2.98300 | -6.77800 |
| O | 28.18400 | -2.63000 | -7.92100 |
| N | 27.97300 | -2.28500 | -5.70100 |
| C | 28.24100 | -2.54000 | -4.37700 |
| N | 27.62600 | -1.77900 | -3.46000 |
| N | 29.06300 | -3.48300 | -3.95400 |
| C | 29.60100 | -4.15800 | -4.98200 |
| C | 23.42900 | 2.94400 | -2.72900 |
| N | 24.31900 | 2.01900 | -3.47100 |
| C | 25.18500 | 1.07300 | -2.97600 |
| N | 25.81400 | 0.43500 | -3.92300 |
| C | 25.33400 | 0.99400 | -5.09800 |
| C | 25.65000 | 0.70700 | -6.45200 |
| O | 26.45200 | -0.13600 | -6.86900 |
| N | 24.93400 | 1.50600 | -7.33500 |
| C | 24.02300 | 2.46800 | -6.97000 |
| N | 23.43100 | 3.14100 | -7.97300 |
| N | 23.72200 | 2.74300 | -5.71000 |
| C | 24.40400 | 1.98000 | -4.83400 |
| C | 23.02200 | 3.73200 | -14.20200 |
| N | 23.49300 | 3.19400 | -12.90900 |
| C | 23.18000 | 3.55200 | -11.62600 |
| N | 23.80800 | 2.83500 | -10.72500 |
| C | 24.57800 | 1.95500 | -11.46100 |
| C | 25.46600 | 0.94200 | -11.03400 |

| | | | |
|---|---|---|---|
| O | 25.76400 | 0.60300 | -9.87300 |
| N | 26.03400 | 0.29500 | -12.12500 |
| C | 25.78400 | 0.58000 | -13.44600 |
| N | 26.44900 | -0.17900 | -14.32600 |
| N | 24.95500 | 1.52500 | -13.87100 |
| C | 24.39700 | 2.16200 | -12.81200 |
| C | 30.21300 | -5.13000 | -15.07300 |
| N | 29.46200 | -4.11800 | -14.29400 |
| C | 28.67600 | -3.08500 | -14.73800 |
| N | 28.16400 | -2.38400 | -13.76200 |
| C | 28.64600 | -2.99800 | -12.61000 |
| C | 28.42100 | -2.67100 | -11.24600 |
| O | 27.72800 | -1.74100 | -10.80900 |
| N | 29.08300 | -3.52900 | -10.38100 |
| C | 29.86600 | -4.57800 | -10.80600 |
| N | 30.42500 | -5.29800 | -9.82700 |
| N | 30.08700 | -4.89900 | -12.07600 |
| C | 29.44800 | -4.07000 | -12.92500 |
| H | 30.65900 | -4.96100 | -2.75700 |
| H | 31.45900 | -6.39200 | -6.29100 |
| H | 27.00800 | -1.06800 | -3.74500 |
| H | 27.79300 | -1.93100 | -2.50200 |
| H | 22.70600 | 3.35800 | -3.40000 |
| H | 25.32200 | 0.88900 | -1.97000 |
| H | 23.65900 | 2.93200 | -8.90800 |
| H | 22.77000 | 3.84200 | -7.77200 |
| H | 23.01900 | 2.95100 | -14.93300 |
| H | 22.51800 | 4.30800 | -11.39100 |
| H | 26.32400 | -0.03700 | -15.29200 |
| H | 27.06300 | -0.87900 | -14.00700 |
| H | 30.40500 | -5.98400 | -14.45700 |
| H | 28.50700 | -2.88300 | -15.73600 |

| | | | |
|---|---|---|---|
| H | 31.00000 | -6.06600 | -10.04800 |
| H | 30.26200 | -5.06000 | -8.88600 |
| H | 25.09400 | 1.37000 | -8.31300 |
| H | 27.33300 | -1.54600 | -5.91000 |
| H | 28.98600 | -3.37800 | -9.39700 |
| H | 26.68100 | -0.44200 | -11.93200 |
| H | 30.49800 | -6.61500 | -3.29600 |
| H | 32.01100 | -5.76900 | -3.51300 |
| H | 29.63500 | -5.42600 | -15.92300 |
| H | 31.14100 | -4.71100 | -15.40100 |
| H | 22.03100 | 4.11800 | -14.08800 |
| H | 23.67600 | 4.51700 | -14.52100 |
| H | 22.92700 | 2.40800 | -1.95000 |
| H | 24.01200 | 3.73300 | -2.30100 |
